# The Pathogenic Aspects of Human Parvovirus B19 NS1 Protein in Chronic and Inflammatory Diseases

**DOI:** 10.1155/2022/1639990

**Published:** 2022-06-06

**Authors:** Sedigheh Jalali, Ali Farhadi, Gholamreza Rafiei Dehbidi, Shirin Farjadian, Sedigheh Sharifzadeh, Reza Ranjbaran, Noorossadat Seyyedi, Sepide Namdari, Abbas Behzad-Behbahani

**Affiliations:** ^1^Diagnostic Laboratory Sciences and Technology Research Center, School of Paramedical Sciences, Shiraz University of Medical Sciences, Shiraz, Iran; ^2^Department of Medical Biotechnology, School of Paramedical Sciences, Shiraz University of Medical Sciences, Shiraz, Iran; ^3^Department of Immunology, School of Medicine, Shiraz University of Medical Sciences, Shiraz, Iran

## Abstract

**Background:**

The nonstructural protein (NS1) of human parvovirus B19 (hPVB19) is considered to be a double-edged sword in its pathogenesis. NS1 protein promotes cell death by apoptosis in erythroid-lineage cells and is also implicated in triggering and the progression of various inflammation and autoimmune disorders.

**Objectives:**

We investigated the possible role of hPVB19 NS1 in the modulation of proinflammatory cytokines in nonpermissive HEK-293T cells.

**Methods:**

A plasmid containing the fully sequenced NS1 gene (pCMV6-AC-GFP-NS1) was transfected into HEK-293T cells. Transfection efficiency was assessed by fluorescent microscopy over time. Mock (pCMV6-AC-GFP) transfected cells were used as controls. The percentage of apoptotic cells was measured by flow cytometry at 24, 48, and 72 h posttransfection. Interleukin 6 (IL-6) mRNA, as a pleiotropic cytokine, was measured by real-time PCR. Furthermore, cellular supernatants were collected to determine the type and quantity of cytokines produced by mock- and NS1-transfected cells using flow cytometry.

**Results:**

Fold change in the expression level of IL-6 mRNA in transfected cells after 72 hr of incubation was found to be 3.01 when compared with mock-transfected cells; however, cell apoptosis did not happen over time. Also, the concentration of cytokines such as IL-2, IL-6, IL-9, IL-17A, IL-21, IL-22, interferon (IFN)-*γ*, and tumor necrosis factor *α* (TNF-*α*) increased in NS1-transfected cells.

**Conclusions:**

Overall, our results indicated that proinflammatory cytokine levels had increased following the expression of hPVB19 NS1 in HEK-293T cells, consistent with a role for NS1 expression facilitating the upregulation of inflammatory reactions. Therefore, hPVB19 NS1 function may play a role in the progression of some chronic and inflammatory diseases.

## 1. Introduction

Human parvovirus B19 (hPVB19) is a small nonenveloped virus with a single-stranded DNA genome that is a known causative agent for erythema infectiosum disease in children. At 2–5 years of age, its seroprevalence rate is reported to be 5–10%. However, it increases to 50% by 15 years of age and 60% by 30 years of age [1, 2]. The incidence of infection in adults over 60 years of age is approximately 90%, with a small percentage of adults acquiring the infection every year. Common symptoms of hPVB19 infection are fever (15–30% of patients), malaise, headache, myalgia, nausea, and rhinorrhea, typically beginning 5–7 days after initial infection [3]. The virus, which has a unique tropism for human erythroid progenitor cells (EPCs), requires the P blood antigen (globoside; Gb4Cer) as a receptor to enter cells [4, 5]. However, two membrane proteins, *α*5*β*1 integrin and Ku80, have also been proposed as possible co-receptors triggering virus entry through endocytosis [6, 7].

As the Gb4Cer receptor is widely expressed, hPVB19 can infect several different cell types. Thus, the virus can be found in a range of tissues and organs. For this reason, the spectrum of hPVB19 associated with a variety of clinical diseases has increased in recent years. Various HPVB19-related diseases include autoimmune disorders [8, 9], carcinomas [9–11], vasculitis [12, 13], hepatitis [14], acute and chronic inflammatory diseases [15–17], and some thyroid cancers [18, 19]. However, little is known about how hPVB19 causes those diseases or which viral protein (s) are responsible for the cytopathic effects and host responses that are triggered by a viral infection.

The hPVB19 viral DNA encodes three major proteins, namely, the nonstructural protein 1 (NS1), and the viral capsid proteins VP1 and VP2 [20]. The disease outcomes following hPVB19 infection are partly due to direct cytotoxicity which is associated with the expression of the NS1 protein [21]. However, inflammatory processes are also related to hPVB19 -induced pathogenesis.

Inflammatory responses are generally regarded as beneficial and protective against infections [22]. However, chronic inflammation can induce tissue damage and has also been linked with the development of some cancers [23]. The hPVB19 NS1 protein is essential for the replication of viral DNA but also acts as a transactivator to trigger signalling cascades that eventually lead to the activation of the proinflammatory cytokine interleukin-6 (IL-6). However, prolonged synthesis of IL-6 has a pathological effect on chronic inflammation and can predispose patients to the development of malignancies and autoimmune diseases [21, 24, 25]. Although cytokines have been implicated in contributing to the pathogenesis of hPVB19 infection, the precise mechanism/s by which they do this are still unclear [13].

Two significant sources of cytokine production are T helper (Th) 1 and Th2 subsets [26, 27]. Th1 cytokines, including IL-2, IFN-*γ*, and TNF-*α*, play essential roles in activating cellular immunity, while Th2 cytokines, such as IL-4, IL-5, IL-6, and IL-13, contribute to regulating humoral immunity [14, 27]. Besides, nonimmune cell types, such as epithelial cells, can release cytokines in response to infections or tissue damage [28]. Overall, NS1 is a multifunctional protein and plays various roles during hPVB19 infection [29].

Given the importance of epithelial cells in first-line responses to a range of pathogens, we reasoned that they represent an appropriate cell type for investigating cellular responses associated with the expression of hPVB19 NS1 protein. In the present study, HEK-293T cells were transfected with a plasmid vector containing the complete sequence of the hPVB19 NS1 gene to examine the effect of NS1 expression on the modulation of cellular cytokine production.

## 2. Materials and Methods

### 2.1. Expression Vector with hPVB19 NS1 Sequence

The full length of a synthetic gene encoding hPVB19 NS1 protein (NCBI Reference Sequence: NC_000883.2) was received in the pCMV6-AC-GFP-NS1 constitutive expression vector (Biomatik, Ontario, Canada). For further analysis, EcoR1 and Xho1 restriction sites were introduced at the 5′ and the 3′ ends of the NS1 gene, respectively. The pCMV6-AC-GFP expression vector was used as a negative control (mock). Lyophilized vectors were reconstituted according to the manufacturer's instructions, and DNA concentrations were measured in triplicate using a Nanodrop spectrophotometer (Thermo Scientific).

### 2.2. PCR Amplification

To verify the NS1 open reading frame (ORF) in the expression vector, conventional PCR was carried out using appropriate forward (5′ATGGAGCTATTTAGAGGGGTGC3′) and reverse (5′TTACTCATAATCTACAAAGCTTTGC3′) primers to amplify a 2016 bp fragment corresponding to the complete sequence of the NS1 gene. The PCR mixture in a final volume of 50 *μ*L contained 5 *μ*L of 10X Pfu buffer with MgSO4 (Thermo Scientific), 5 *μ*L of 200 *μ*M dNTP mix, 0.5 *μ*M of each primer, 1 *μ*L plasmid DNA (50 pg), and Pfu DNA polymerase 1.5 U (Thermo Scientific), and the sample was made up to the final volume with PCR grade double-distilled water. PCR was performed using the following conditions: 5 min at 95°C, followed by 32 cycles of 95°C for 50 s, 58°C for 1 min, and 72°C for 5 min, followed by 72°C for 6 min. For final confirmation, PCR products were gel purified and sequenced.

### 2.3. Cell Culture and Transfection

HEK-293T cells (ATCC CRL-3216) were grown in Dulbecco's Modified Eagle Medium (DMEM) supplemented with 10% fetal bovine serum (FBS) (Gibco-BRL, USA) and 1% penicillin-streptomycin (10.000 U/ml) at 37°C in 5% CO_2_. 1 × 10^6^ cells in 6-well culture plates were transfected with 2.5 *μ*g/*μ*l of each pCMV6-AC-GFP-NS1 or pCMV6-AC-GFP vectors, using lipofectamine 3000 (Invitrogen, CA, USA, Catalogue number: L3000001) according to the manufacturer's instructions. Cells were then cultured in serum-free DMEM for 12 h at 37°C in 5% CO_2_ and subsequently in DMEM with 10% FBS. Transfection efficiency and expression of GFP were assessed by inverted fluorescence microscopy (Hund, Germany) at 24 hr, 48 hr, and 72 hr posttransfection.

### 2.4. Apoptosis Assay

An apoptosis assay was performed on mock- or NS1-transfected cells using the PE Annexin V apoptosis detection kit according to the manufacturer's instructions (BD PharMingen, San Diego, CA, Catalogue number: 559763). PE Annexin V was used to quantitatively determine the percentage of cells undergoing the early phases of apoptosis through bounding to phospholipid phosphatidylserine. In this kit, 7-AAD is also used as a vital dye to distinguish viable from nonviable cells. Cells were analyzed using a FACSCalibur flow cytometer (eBioscience, Santa Cruz, CA, USA). Cells that stained double-positive for PE Annexin V and 7-AAD were considered as apoptotic cells.

### 2.5. RNA Extraction and cDNA Synthesis

Total RNA was extracted from mock- and pCMV6-AC-GFP-NS1-transfected HEK-293T cells at 24, 48, and 72 hr posttransfection using TRIzol reagent (Invitrogen, Germany, Catalogue number: 15596026) according to the manufacturer's instructions. cDNA was synthesized using the PrimeScript First strand cDNA synthesis kit (Takara, Japan, Catalogue number: 6110A) according to manufacturer's instructions. To remove any contaminating genomic DNA, the extracted RNA was treated with 5 U of amplification grade DNase I (Invitrogen, Carlsbad, CA, Catalogue number: 18068015) according to the manufacturer's instructions. The concentration and purity of the extracted RNA were measured using a Nanodrop spectrophotometer (Thermo Scientific).

### 2.6. Real-Time Quantitative qRT-PCR

RT-qPCR was performed using a Rotor-Gene Q 5-plex thermal cycler system (Qiagen, Hilden, Germany) and SYBR Green real-time PCR master mix (Takara, Japan, Catalogue number: 639676) according to the manufacturer's guideline. Furthermore, the housekeeping *β* actin gene was tested as a control for cDNA quantity and quality. For the amplification of IL-6, reaction mixtures contained 12.5 *μ*L of Takara SYBR Green, 0.6 *μ*L of each primer (0.5 *μ*M), 2 *μ*L of cDNA (50 ng/*μ*l), and 9.3 *μ*L of PCR grade water. Tubes were then incubated for 1 min at 94°C, followed by 40 cycles of 94°C for 30 s, 58°C for 30 s, and 72°C for 30 s. A standard curve was generated to estimate amplification efficiency. Using specific primers, the same RT-PCR conditions were used for the detection and quantification of hPVB19 NS1 mRNA in the samples. The sequences of primers used in the reactions are shown in Table 1.

### 2.7. Cytokine Assay

Cell culture supernatants were collected at 24, 48, and 72 h posttransfection and analyzed simultaneously for 13 different human T-helper (Th) cytokines (IL-2, IL-4, IL-5, IL-6, IL-9, IL-10, IL-13, IL-17A, IL-17F, IL-21, IL-22, IFN-*γ*, and TNF-*α*) using a multiplex bead-based assay with a commercial kit (BioLegend, San Diego, CA, USA, Catalogue number: 740721). The procedure was performed according to the manufacturer's instructions. Briefly, antibody-coated beads for each cytokine, which could be differentiated by their sizes and fluorochrome intensities, were incubated with supernatants or standards. Then, the secondary antibody conjugated to biotin was added, followed by adding PE-conjugated streptavidin. Data were analyzed using flow cytometry and the FlowCytomix Pro-3.0 software (eBioscience).

### 2.8. Statistical Analysis

A paired *t*-test was used to compare differences between NS1-transfected and untransfected cells. All statistical analyses were done with SPSS version 18 (SPSS Inc., Chicago, IL, USA), and a two-sided *P* < 0.05 was considered statistically significant. GraphPad Prism 5 (La Jolla, CA, USA) was used to design all charts.

## 3. Results

### 3.1. Expression of GFP and B19 NS1 in Transfected HEK-293T

**Figure 1:**
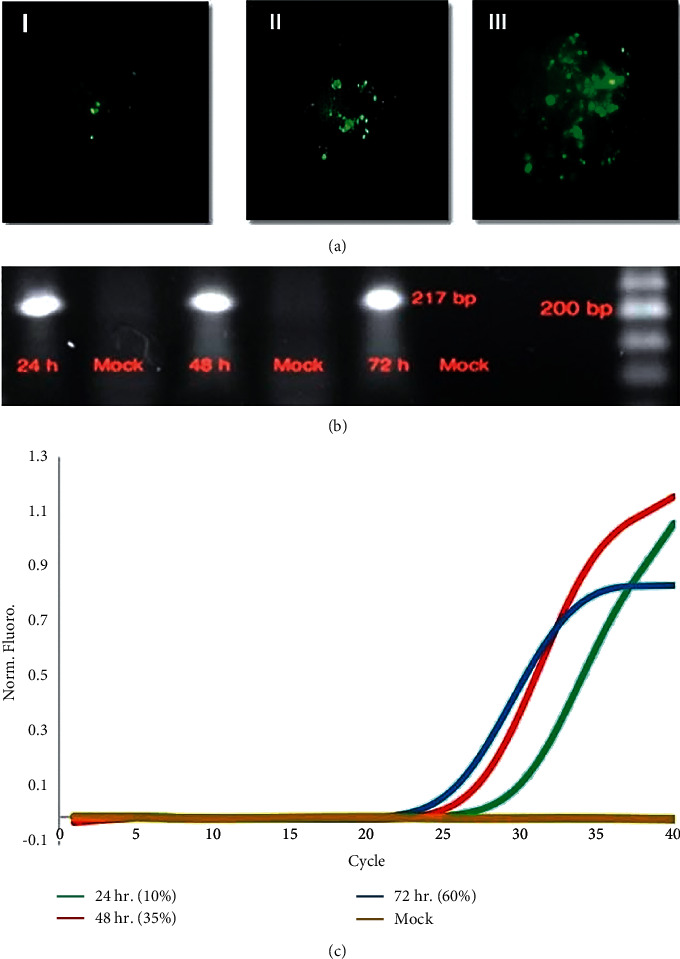
(a) Expression of GFP in NS1-transfected HEK-293T cells was examined by fluorescence microscopy at (I) 24 hr (II) 48 hr (III) 72 hr posttransfection with 10x magnification and bar size of 25 *μ*m. (b) The expression of NS1 mRNA in HEK-293 T cells was examined and visualized on 1.5% agarose gel at 100 V for 40 min and stained with GelRed at the indicated times posttransfection. (c) Posttransfection expression levels of NS1 mRNA overtime. Green: after 24 hr, red: after 48 hr, blue: after 72 hr, brown: mock-transfection at 24, 48, and 72 hr posttransfection.

1 × 10^6^ HEK-293T cells were transfected with equal amounts of expression vectors encoding the hPVB19 NS1 gene or with the control vector (mock). Transfection efficiency was monitored by fluorescence microscopy at 24, 48, and 72 hr posttransfection (Figure 1(a)) with the number of transfected cells and the intensity of GFP fluorescence increasing over time. Using Lipofectamine 3000, transfection efficiency for GFP in HEK-293S cells was ∼10% (28), ∼35% (25.5), and ∼60% (24) at 24, 48, and 72 hr posttransfection. GFP fluorescence was at its highest level 3 days after transfection. The expression of NS1 mRNA in HEK-293 T cells was confirmed and visualized on agarose gel (Figure 1(b)). Real-time RT-PCR analysis of NS1 mRNA expression demonstrated a time-dependent increase in NS1 mRNA after HEK-293T cell transfection with a plasmid containing the HPVB19NS1 gene (Figure 1(c)).

### 3.2. Quantification of IL-6 mRNA by RT-qPCR

**Figure 2:**
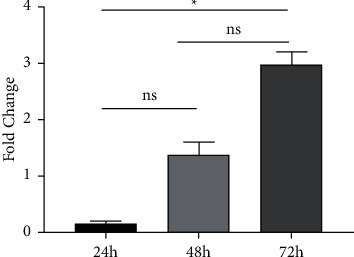
Fold change of IL-6 mRNA transcription in HEK-293T cells transfected with mock and B19NS1 vectors overtime in four experiments.

Next, we examined the levels of IL-6 transcripts in cells with HPVB19NS1 vector or the control vector (mock) at various times after transfection. Our results showed an increasing trend in IL-6 transcripts in NS1-transfected cells compared to mock-transfected cells (Figure 2). Fold change in the expression level of IL-6 in transfected cells after 72 hr of incubation was found to be 3.01 when compared with mock-transfected cells. However, 24 hr after transfection, this ratio was quite low (0.61).

### 3.3. Evaluation of Apoptosis in NS1-Transfected Cells

**Figure 3:**
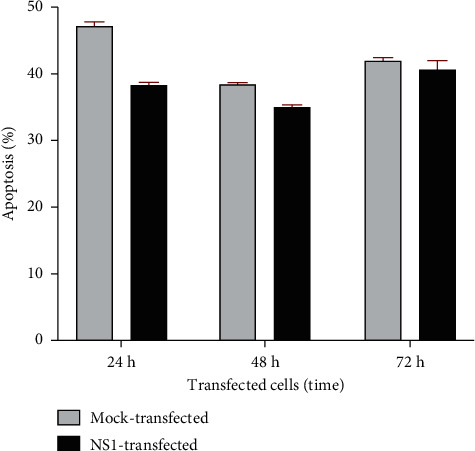
Percentage of apoptotic cells in mock- and NS1-transfected HEK-293T cells at 24 hr, 48 hr, and 72 hr posttransfection was determined by flow cytometry of cells that stained double-positive for PE Annexin V and 7-AAD.

To evaluate the potency of NS1 expression in inducing cell death, apoptotic cells were measured by flow cytometry. When compared with mock-transfected cells, no statistically significant difference is observed between test and control samples (*P*=0.1728) (Figure 3).

### 3.4. Quantification of Cytokines in Culture Supernatants by Flow Cytometry

Using a multiplex bead-based assay, culture supernatants from HEK-293T cells that had been mock- or B19 NS1-transfected were collected to measure the levels of 13 different cytokines. Supernatants from untransfected control cells were also included for analysis (Table 2). Cytokine concentrations in samples were quantitated using standard curves according to the manufacturer's instruction.

**Figure 4:**
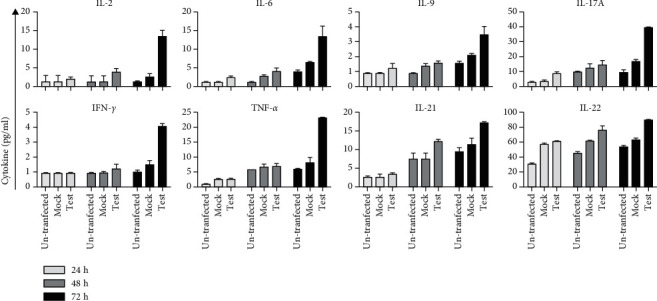
Increases in the levels of proinflammatory cytokines over time. No significant differences were found between the concentration of IL-2, IL-6, IL-9, and INF*γ* in 24 to 48 hours after transfection in different test groups, whereas, the differences between the concentration of IL-17A and TNF-*α* among the groups was significant (*P* < 0.0006).

Overall, using a 2way ANOVA Tukey's multiple comparisons test, the concentrations of proinflammatory cytokines, such as IL-2, IL-17A, IFN-*γ*, TNF-*α*, and IL-6, were found to have increased significantly (*P* < 0.0001) in supernatants of cells transfected with a plasmid containing the hPVB19 NS1 gene 72 hr after transfection. It is noteworthy that the levels of several anti-inflammatory cytokines (such as IL-4, IL-5, IL-10, and IL-17F) remained unchanged over the various incubation times, indicating that they were not strongly induced following the expression of hPVB19 NS1. Levels of IL-13, a cytokine closely related to IL-4 and produced by Th2 cells, also increased in supernatants of cells transfected to express the B19 NS1 gene (Figure 4). Also, several other cytokines were induced in the supernatants of cells transfected to express hPVB19 NS1. These included: IL-9, a cytokine associated with cell signalling molecule; IL-21, an immunoregulatory cytokine; and IL-22, a potent mediator of cellular inflammatory responses (Table 2).

Paired *t*-test analysis was performed to compare the cytokine concentration means of NS1-transfected cells with untransfected or mock-transfected ones at different time-points. No statistically significant difference was found.

## 4. Discussion

Several studies have suggested that inflammation is likely to be a trigger for cancer development associated with chronic viral infections. Among viruses, hPVB19 is associated with several acute and chronic inflammatory diseases. Although most symptoms appear to relate to the tissue tropism of this virus, the mechanism (s) by which hPVB19 induces inflammatory response is poorly understood. In general, B19 viral replication is under the control of trans-activation by the viral NS1 protein [30, 31]. However, NS1 can also trans-activate a variety of genes, such as IL-6 and TNF-*α* [20, 32]. Apart from the trans-activating function, NS1 can induce cell apoptosis through its cytotoxic effect [33]. In the present study, we have used transfected HEK-293T cells to provide evidence for a role of the viral NS1 protein in the modulation of cellular inflammatory responses.

Analysis of apoptosis at 24, 48, and 72 hr posttransfection demonstrated that relative to mock-transfected cells, transfection of a vector expressing NS1 did not result in enhanced apoptosis.

To examine the impact of NS1 expression on the induction of cytokine responses in HEK-293T cells, we used a flow cytometry-based assay to measure an array of Th1/Th2/Th17/Th9/Th22-related cytokines. We found that NS1 expression was associated with the production of some proinflammatory cytokines, while levels of a number of anti-inflammatory cytokines did not increase. For example, our results demonstrated that higher levels of IL-6 and TNF-*α* were released from NS1-transfected cells compared to mock-transfected and untransfected cells. In agreement with previous reports, we also report the elevated levels of IL-21 and IL-22, consistent with the induction of these cytokines following the production of IL-6 and TNF-*α* through STAT-3 signalling [21, 34].

Th-17 plays an essential role in the induction and/or progression of some autoimmune diseases [35]. IL-17 binds to and signals through IL-17 receptor A (IL-17RA), which is widely expressed on epithelial cells, endothelial cells, and fibroblasts. The ligation of IL-17/IL-17R results in the release of inflammatory mediators like IL-1, IL-6, IL-8, IL-23, TNF-*α*, and several chemokines to further stimulate the inflammatory cascade. Since inflammation is regarded as a promoter to carcinogenesis, evaluating IL-17 in cancer development is a necessity [36]. Moreover, the results of some studies have demonstrated that NS1 is involved in several autoimmune disorders such as Hashimoto's disease, systemic lupus erythematosus (SLE), and rheumatoid arthritis (RA) [6–8]. Our results showed elevated IL-17A levels in the supernatants from NS1-transfected cells.

Previous studies by Kerr et al. [13] reported a significant increase in proinflammatory cytokines in hPVB19-infected patients with RA. In line with these findings, our results showed increased levels of IFN-*γ* and TNF-*α* in supernatants from NS1-transfected cells, because Th1 cytokines such as IFN-*γ* and TNF-*α* can induce IL-6, there could be a potential role for NS1 in triggering various inflammatory and autoimmune disorders that have been linked to hPVB19 infections.

We also reported an increase in IL-9 production by NS1-transfected HEK-293T cells. It has been reported that IL-9 production was associated with delayed clearance of respiratory syncytial virus (RSV). IL-9 is a previously unknown but key modulator of antiviral immunity, regulating T and B cell responses, and having potent and specific effects on viral lung disease [37].

Additionally, IL-2 levels were elevated in cells transfected with NS1. IL-2 contributes to a variety of autoimmune disorders, including the production of regulatory T cells, which suppress autoimmune disease [38]. With these dual roles of IL-2, it should be considered in a more complex context of cytokine network.

In this study, the NS1 gene from human B19 virus was examined for its effects on cell function. It is also possible to create chimeric genes using molecular techniques to study how other genes affect cell function [39].

## 5. Conclusion

In general, our study showed that human parvovirus B19 NS1 gene expression in the cell was associated with higher levels of several proinflammatory cytokines. However, the concentration of specific anti-inflammatory cytokines was not altered compared with a mock-transfected cell population. This study suggests that the human parvovirus B19 NS1 protein may be a double-edged sword in viral pathogenesis. To investigate the possibility that the B19 virus NS1 gene causes inflammation or prevents apoptosis, we must use different cell lines representing specific tissues with specific receptors for the virus.

## Figures and Tables

**Table 1:** Primers used for PCR analysis.

Primers	Sequence
IL-6-forward	5′-CCTCACCTCTTCCATTCC-3′
IL-6-reverse	5′-TATCACCTCCACGCTCAA-3′
*β*-Actin-forward	5′-GCACAGAGCCTCGCCTTT-3′
*β*-Actin-reverse	5′-GCCTCGTCGCCCACATAG-3′
NS1-forward	5′-AGACACCAGTATCAGCAGCA-3′
NS1-reverse	5′-TGCCAAAGGTGTGTAGAAGG-3′

**Table 2:** Thirteen cytokines' concentration of mock-transfected, B19 NS1-transfected, and untransfected HEK-293T cell are measured at 24, 48, and 72 hr posttransfection. Cytokine concentrations are reported based on a picogram per milliliter as mean. The experiments were carried out two times in duplicate.

Posttransfection time (h)	HEK-293T	Cytokines (pg/ml) mean ± SD												
		IL-13	IL-10	IL-4	IL-2	IL-6	IL-9	IFN-*γ*	TNF-*α*	IL-17A	IL-17F	IL-21	IL-22	IL-5
24	Untransfected cell	1.21 ± 0.19	1.67	1.37	1.25 ± 1.18	1.15 ± 0.68	1.4 ± 0.43	0.95 ± 0.02	0.88 ± 0.02	3.42 ± 0.2	1.24	2.62 ± 0.16	43.8 ± 0.3	0.64
	Mock-transfected	1.21 ± 0.19	1.67	1.37	1.25 ± 1.18	2.61 ± 0.09	1.64 ± 0.1	1.02 ± 0.02	5.98 ± 0.5	7.88 ± 1.0	1.24	6.86 ± 1.7	63.6 ± 0.9	0.64
	NS1- transfected	1.72 ± 0.35	1.67	1.37	4.37 ± 0.19	8.56 ± 3.56	2.6 ± 1.44	1.02 ± 0.02	7.44 ± 0.7	16.47 ± 1.5	1.24	8.58 ± 2	72.48 ± 3.0	0.64

48	Untransfected cell	1.21 ± 0.13	1.67	1.37	1.25 ± 1.12	2.26 ± 0.78	1.26 ± 0.28	0.95 ± 0.02	5.81 ± 0.5	3.90 ± 0.24	1.24	1.98 ± 0.2	30.42 ± 0.4	0.64
	Mock- transfected	1.21 ± 0.13	1.67	1.37	1.25 ± 1.12	3.99 ± 1.66	1.68 ± 0.19	0.95 ± 0.02	6.15 ± 0.3	4.25 ± 0.1	1.24	3.17 ± 1.4	61.01 ± 0.7	0.64
	NS1- transfected	1.67 ± 0.14	1.67	1.37	1.99 ± 0.35	11.23 ± 0.71	2.46 ± 1.9	0.95 ± 0.02	6.32 ± 1.0	18.8 ± 1.1	1.24	12.58 ± 0.18	62.88 ± 1.2	0.64

72	Untransfected cell	1.52 ± 0.32	1.67	1.37	1.25 ± 0.16	3.12 ± 0.42	1.7 ± 0.14	1.09 ± 0.09	6.01 ± 0.4	10.48 ± 1	1.24	10.15 ± 1.2	53.30 ± 2.1	0.64
	Mock- transfected	1.67±	1.67	1.37	2.62 ± 0.58	8.47 ± 1.73	2.12 ± 0.1	1.32 ± 0.1	7.69 ± 0.6	14.29 ± 1.5	1.24	12.58 ± 0.5	56.71 ± 1.3	0.64
	NS1- transfected	5.25 ± 2.15	1.67	1.37	13.50 ± 1.13	14.46 ± 2.09	4.65 ± 1.2	4.94 ± 0.8	23.49 ± 0.3	39.56 ± 1.2	1.24	17.71 ± 0.24	90.23 ± 3	0.64

## Data Availability

Data are available within the article.
